# Novel Curcumin C66 That Protects Diabetes-Induced Aortic Damage Was Associated with Suppressing JNK2 and Upregulating Nrf2 Expression and Function

**DOI:** 10.1155/2018/5783239

**Published:** 2018-11-28

**Authors:** Cheng Li, Xiao Miao, Shudong Wang, Binay Kumar Adhikari, Xin Wang, Jian Sun, Quan Liu, Qian Tong, Yonggang Wang

**Affiliations:** ^1^Department of Cardiovascular Center, The First Hospital of Jilin University, Changchun, Jilin 130021, China; ^2^The Second Hospital of Jilin University, Changchun, Jilin 130000, China; ^3^Department of Thyroid Surgery, The First Hospital of Jilin University, Changchun, Jilin 130021, China

## Abstract

Diabetes-related cardiovascular diseases are leading causes of the mortality worldwide. Our previous study has explored the protective effect of curcumin analogue C66 on diabetes-induced pathogenic changes of the aorta. In the present study, we sought to reveal the underlying protective mechanisms of C66. Diabetes was induced in male WT and JNK2^−/−^ mice with a single intraperitoneal injection of streptozotocin. Diabetic mice and age-matched nondiabetic mice were randomly treated with either vehicle (WT, WT DM, JNK2^−/−^, and JNK2^−/−^DM) or C66 (WT + C66, WT DM + C66, JNK2^−/−^ + C66, and JNK2^−/−^DM + C66) for three months. Aortic oxidative stress, cell apoptosis, inflammatory changes, fibrosis, and Nrf2 expression and function were assessed by immunohistochemical staining for the protein level and real-time PCR method for mRNA level. The results suggested that either C66 treatment or JNK2 deletion can reverse diabetes-induced aortic oxidative stress, cell apoptosis, inflammation, and fibrosis. Nrf2 was also found to be activated either by C66 or JNK2 deletion. However, C66 had no extra effect on diabetic aortic damage or Nrf2 activation without JNK2. These results suggest that diabetes-induced pathological changes in the aorta can be protected by C66 mainly via inhibition of JNK2 and accompanied by the upregulation of Nrf2 expression and function.

## 1. Introduction

Cardiovascular diseases are associated with a substantial morbidity and mortality worldwide. People with diabetes mellitus exhibit a higher risk of cardiovascular diseases compared with that of the general population. Cardiovascular complications caused more than a half of death in diabetic patients [1, 2]. There is a prediction that the number of diabetic patients reaches almost 600 million by 2035 [3]. Hyperglycemia causes irreversible damage to blood vessels by inducing both micro- and macrovascular complications in various organs like the skin, muscles, heart, brain, eyes, and kidneys [4, 5]. Poor control of blood glucose at the early stage of diabetes has been demonstrated to accelerate the incidence and progression of vascular damage. Therefore, new therapies to prevent diabetic complications should be paid more attention.

There is considerable evidence that hyperglycemia-induced vascular damage is associated with the generation and accumulation of reactive oxygen species, ultimately leading to increased oxidative stress [6, 7]. Oxidative stress exhibits an imbalance between the free radical production and the endogenous physiological antioxidant mechanisms that lead to the activation of stress-sensitive intracellular signaling pathways and increased cellular damage. The damage of endothelial cells contributes to vasoconstriction, cellular proliferation, leukocyte aggregation, thrombosis, and inflammation predisposing to atherosclerosis [8]. It has been reported that the lipid bilayer of endothelia cells can be destroyed by reactive oxygen species, releasing inflammatory and apoptotic cytokines [9]. Moreover, injury to endothelial cells causes collagen exposure, platelet activation, and aggregation at the injury site, thus contributes to a cascade of thrombosis and inflammation [10]. It is believed that oxidative stress and inflammation are reciprocal causes and outcomes. Nuclear factor (erythroid-derived 2)-like 2 (Nrf2) plays a crucial role in the regulation of the environmental stress by inducing the expression of detoxification and antioxidant enzymes. Under unstressed condition, Nrf2 can be ubiquitinated and degraded by its inhibitor Kelch-like ECH-associated protein 1 (Keap1). When cells are exposed to oxidative stress, Nrf2 can dissociate from Keap1 and translocate to the nucleus, leading to its activation [7]. Once Nrf2 is activated, it regulates the expression of a vast number of genes, including those genes that regulate antioxidants and detoxification enzymes as well as inflammatory responses [11]. It has been shown that the activation of Nrf2 can reduce oxidative stress and inflammation in diabetes, while its absence can aggravate the diabetic complications [12, 13]. Therefore, the activation of Nrf2 may be particularly helpful in combating the deleterious effects of hyperglycemic stress.

The c-Jun N-terminal kinases (JNKs) can be activated by a range of stimuli and were known as “stress-activated protein kinases”. JNKs, belonging to the mitogen-activated protein kinase superfamily, play a crucial role in stress responses, cell survival, and apoptosis [14]. There are three isoforms: JNK1, JNK2, and JNK3; JNK1 and JNK2 are ubiquitously expressed, and JNK3 expresses strictly in the brain, heart, and testis [15].

It has been reported that JNK2 is associated with hypercholesterolemia-induced endothelial dysfunction and oxidative stress and is required for foam cell formation within the atherosclerotic plaque [16, 17]. And JNK2 isoform has shown a more prominent role in the development of obesity-associated insulin resistance [18]. Moreover, deletion of JNK2 has been demonstrated to block diabetic-induced protein nitroxylation [19]. Thus, we speculate that JNK2 plays an important role in diabetes-induced aortic damage.

Curcumin, a natural compound, is the most active agent of the polyphenolic curcuminoids derived from the root of turmeric (*Curcuma longa*). Traditionally, it has been widely used as an herbal medicine and/or food flavoring. Recently, compelling studies show the protective effect of curcumin on human health through its anti-inflammatory, antioxidant, and antimicrobial properties [20–22]. Therefore, curcumin and its analogues have attracted extensive attention. Several studies have reported that (2E,6E)-2,6-bis[2-(trifluoromethyl)benzylidene]cyclohexanone (so-called compound C66), a novel curcumin, has memorable effects in diabetes-related complications based on its anti-inflammatory, antifibrotic, antioxidative, and antiapoptotic properties [23–25]. These studies have also demonstrated that the C66 protection in diabetes is accompanied by inhibition of JNK function. A molecular docking predicted that C66 may target JNK2, which leads to its protective properties [26]. Therefore, we use JNK2 gene knockout mice to verify that the protection of C66 on diabetes-induced aortic damage is associated with inhibition of JNK2.

## 2. Material and Methods

### 2.1. Animals

JNK2^−/−^ and wild-type (WT) male mice on B6.129S2-Mapk9tm1Flv/J genetic background, 6-8 weeks of age, were purchased from the Jackson Laboratory (Bar Harbor, ME, USA). Animals were housed in the Animal Center of Jilin University at a constant room temperature with a 12 : 12 light-dark cycle. A standard rodent diet and water were provided. All animals were acclimatized to the environment for 1 week before being used. Type 1 diabetic mouse model was established by intraperitoneal injection of STZ ((Sigma-Aldrich, St. Louis, MO, USA), dissolved in 0.1 M sodium citrate buffer (pH 4.5)) at 150 mg/kg, while the control animals received the same volume of sodium citrate buffer. Three days after STZ injection, the blood glucose was tested by a glucometer, and the blood glucose levels ≥ 250 mg/dl were considered as diabetic (DM). Then, both diabetic WT mice and JNK2^−/−^mice were randomly divided into two groups: WT DM (*n* = 8) and C66-treated WT DM (WT DM + C66, *n* = 8) and JNK2^−/−^ DM (*n* = 8) and C66-treated JNK2^−/−^ DM (JNK2^−/−^DM + C66, *n* = 8). The age-matched control WT and JNK2^−/−^ mice were also randomly divided into two groups, respectively: WT (*n* = 8) and C66-treated WT (WT + C66, *n* = 8) and JNK2^−/−^ (*n* = 8) and C66-treated JNK2^−/−^ (JNK2^−/−^ + C66, *n* = 8). In the four C66-treated groups, mice were orally administered C66 at 5 mg/kg once a day in alternating days for 3 months, while the four matched groups were given 1% CMC-Na solution alone according to the same schedule.

### 2.2. Aorta Preparation and Histology Staining

Animals were executed after anesthesia, and the thoracic aortas were isolated. Aortic tissues were fixed in 4% paraformaldehyde for more than 24 h, and then they were dehydrated and paraffin-embedded. The fixed tissues were cut into 4 *μ*m thick sections for Masson's trichrome staining and immunohistochemical staining.

For immunohistochemical staining, the tissue slices were deparaffinized by dimethylbenzene, dehydrated by graded ethanol, and then microwaved for 10 min in 1% PBS buffer (pH 7.4, Sangon Biotech Inc., Shanghai, China) for antigen retrieval. When the tissue slices were cooled at room temperature, they were washed with 1% PBS three times and infiltrated with 0.1% Triton X-100 for 15 min. In order to block endogenous peroxidase, all tissue slices were incubated with 3% hydrogen peroxide for 10 min in the dark. The tissue slices were incubated with 10% goat serum for 60 min at 37°C and then with primary antibodies (anti-MCP-1 1 : 250, anti-TNF-*α* 1 : 300, anti-CTGF 1 : 150, anti-TGF-*β*1 1 : 200, anti-HO-1 1 : 150, and anti-SOD-1 1 : 50 (Abcam Inc., America)) at 4°C overnight. Thereafter, all sections were incubated with secondary antibodies (HRP-labeled goat anti-rabbit IgG (H + L) 1 : 400 or HRP-labeled goat anti-mouse IgG (H + L) 1 : 400 (Beyotime Inc., Shanghai, China)) for 40 min at 37°C and then stained with DAB.

For immunofluorescent staining, after antigen retrieval and 0.1% Triton X-100 infiltration, primary antibody anti-Nrf2 was used at 4°C overnight. Thereafter, all sections were incubated with secondary antibody (Cy3-labeled goat anti-rabbit IgG (H + L) (Beyotime Inc., Shanghai, China)) for 40 min at 37°C and then stained with DAPI. A negative control was performed just by incubating with secondary antibody.

### 2.3. Terminal Deoxynucleotidyl Transferase-Mediated dUTP Nick End Labelling (TUNEL) Staining

TUNEL staining was performed with formalin-fixed and paraffin-embedded sections using TUNEL staining kit (DeadEnd™ Colorimetric TUNEL System, Promega Inc., USA) according to the manufacturer's instructions. Positively stained apoptosis cells were counted in at least five random microscopic fields for each slice. Each group had five slices. Cells with TUNEL-positive nuclei were counted under high magnification 400x. The results were presented as TUNEL-positive nuclei per 100 vascular cell nuclei.

### 2.4. Quantitative Real-Time PCR (qRT-PCR)

Total RNA was extracted from aortic tissues using the AxyPrep™ multisource total RNA kit (Axygen Scientific Inc.). RNA was reverse transcribed to cDNA using the TransScript All-in-One First-Strand cDNA Synthesis SuperMix (Transgen Biotech Inc., Beijing, China). Real-time quantitative RT-PCR analysis was carried out using the TransStart Top Green qPCR SuperMix (Transgen Biotech Inc., Beijing, China) and the ABI 7300 Real-Time qPCR System. The primers of CTGF, TGF-*β*1, MCP-1, TNF-*α*, HO-1, SOD-1, and Nrf2 were synthesized by Sangon Biotech (Shanghai, China), and the sequences are listed in Table 1. Data were expressed as number of fold increase compared with levels measured in controls by using the ΔΔ^Ct^ method and *β*-actin as a reference gene.

### 2.5. Statistical Analysis

Variation between different groups was analyzed by one-way analysis of variance (ANOVA) test using Tukey test with Origin 8.0 Lab data analysis. Statistical significance was considered if *P* < 0.05.

## 3. Results

### 3.1. Either JNK2 Deletion or C66 Treatment Can Attenuate Diabetes-Induced Aortic Fibrosis

**Figure 1:**
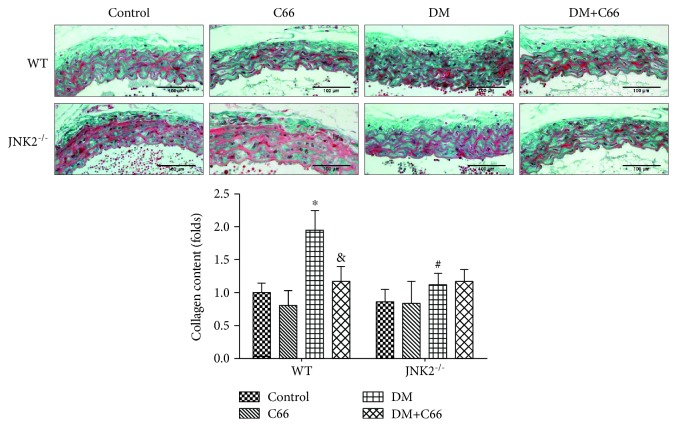
The accumulation of collagen was detected by Masson staining. *n* = 8; ^∗^ *P* < 0.05 DM vs. corresponding control group; ^&^ *P* < 0.05 DM + C66 vs. corresponding DM; ^#^ *P* < 0.05 JNK2^−/−^ mice vs. corresponding WT mice. DM: diabetes mellitus.

At the end of the experiment, the collagen accumulation in tunica media of the aortas was examined by Masson staining (Figure 1). The results showed that C66 treatment or JNK2 deletion can significantly reverse collagen accumulation in the aortas in diabetic mice. However, there was no significant difference between the DM group and DM + C66 group in JNK2^−/−^ mice.

**Figure 2:**
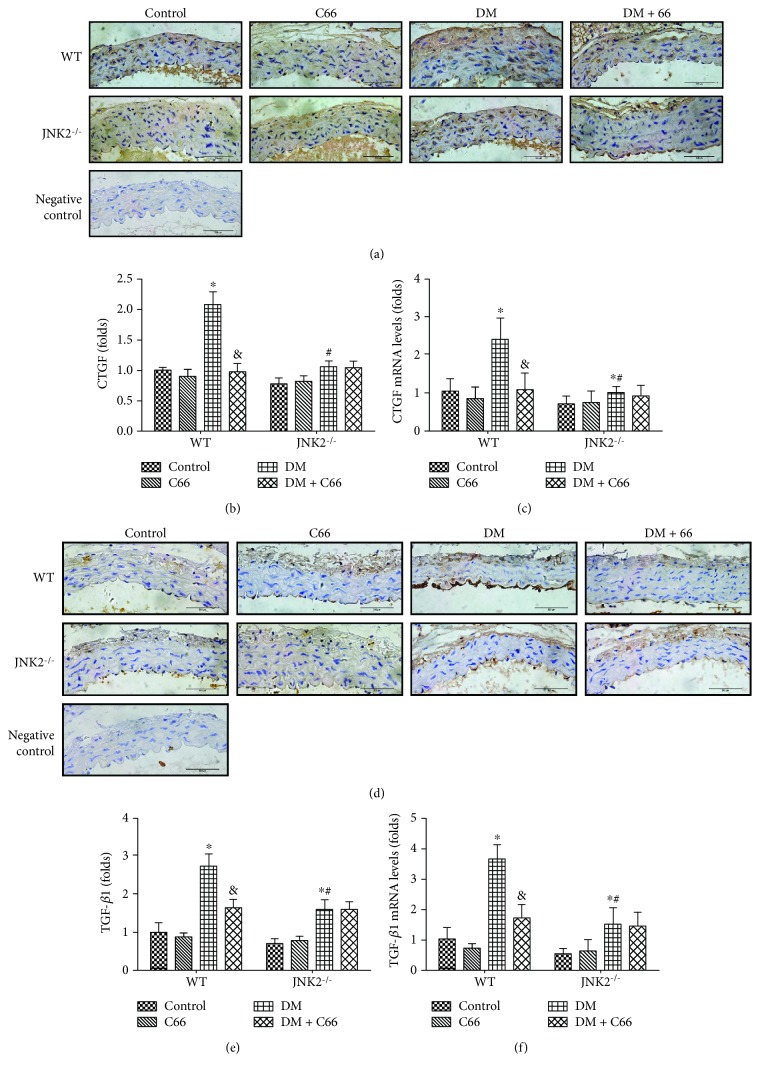
Protective effect of C66 or JNK2 deletion on diabetes-induced aortic fibrosis. Immunohistochemical staining for the expression of CTGF (a, b) and TGF-*β*1 (d, e) and qPCR for the mRNA expression of CTGF (c) and TGF-*β*1 (f). *n* = 8; ^∗^ *P* < 0.05 DM vs. corresponding control group; ^&^ *P* < 0.05 DM + C66 vs. corresponding DM; ^#^ *P* < 0.05 JNK2^−/−^ mice vs. corresponding WT mice. DM: diabetes mellitus.

Immunohistochemical stain was used to evaluate the expression of profibrotic mediators, CTGF (Figure 2(a)) and TGF-*β*1 (Figure 2(d)) in aortic tunica media. Supplementation with C66 or deletion of JNK2 obviously prevented these fibrotic responses in the aortas of diabetic mice (the WT DM + C66 group and JNK2^−/−^ DM group) (Figures 2(b) and 2(e)). Similarly, C66 has not shown its further effect in JNK2^−/−^ DM mice. We also used qPCR to evaluate the mRNA levels of CTGF (Figure 2(c)) and TGF-*β*1 (Figure 2(f)). The results were consistent with the immunohistochemical stain.

### 3.2. Either JNK2 Deletion or C66 Treatment Can Attenuate Diabetes-Induced Aortic Cell Apoptosis

**Figure 3:**
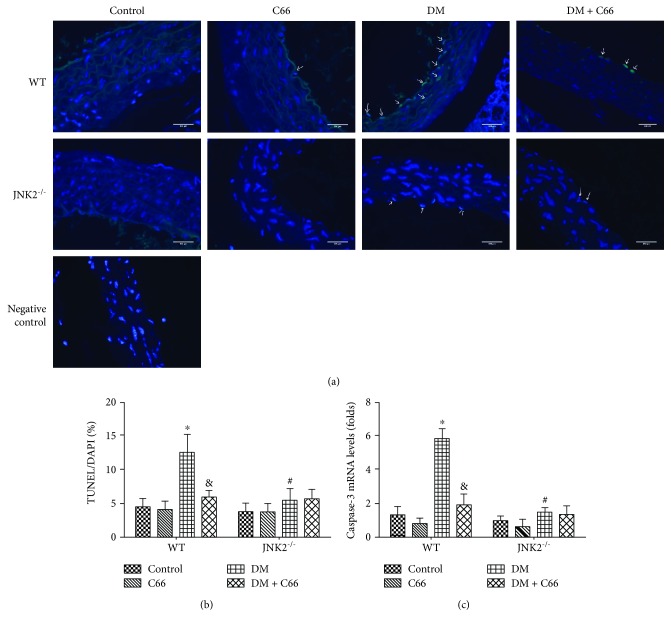
Protective effect of C66 or JNK2 deletion on diabetes-induced aortic apoptosis. The apoptotic cell was examined by TUNEL staining (a, b); the expression of caspase-3 mRNA (c) reflects the apoptotic level. *n* = 8; ^∗^ *P* < 0.05 DM vs. corresponding control group; ^&^ *P* < 0.05 DM + C66 vs. corresponding DM; ^#^ *P* < 0.05 JNK2^−/−^ mice vs. corresponding WT mice. DM: diabetes mellitus.

We used the TUNEL assay to analyze apoptosis of aortic cells (Figure 3(a)). The results showed that diabetes-induced increased apoptosis can be significantly reversed by C66 treatment or JNK2 deletion, but C66 seemed to have no effect on JNK2 deletion diabetic mice (Figure 3(b)). Similar result was also found in the mRNA expression of apoptosis-related protein caspase-3 (Figure 3(c)) via qPCR.

### 3.3. Either JNK2 Deletion or C66 Treatment Can Attenuate Diabetes-Induced Aortic Inflammation and Oxidative Stress

**Figure 4:**
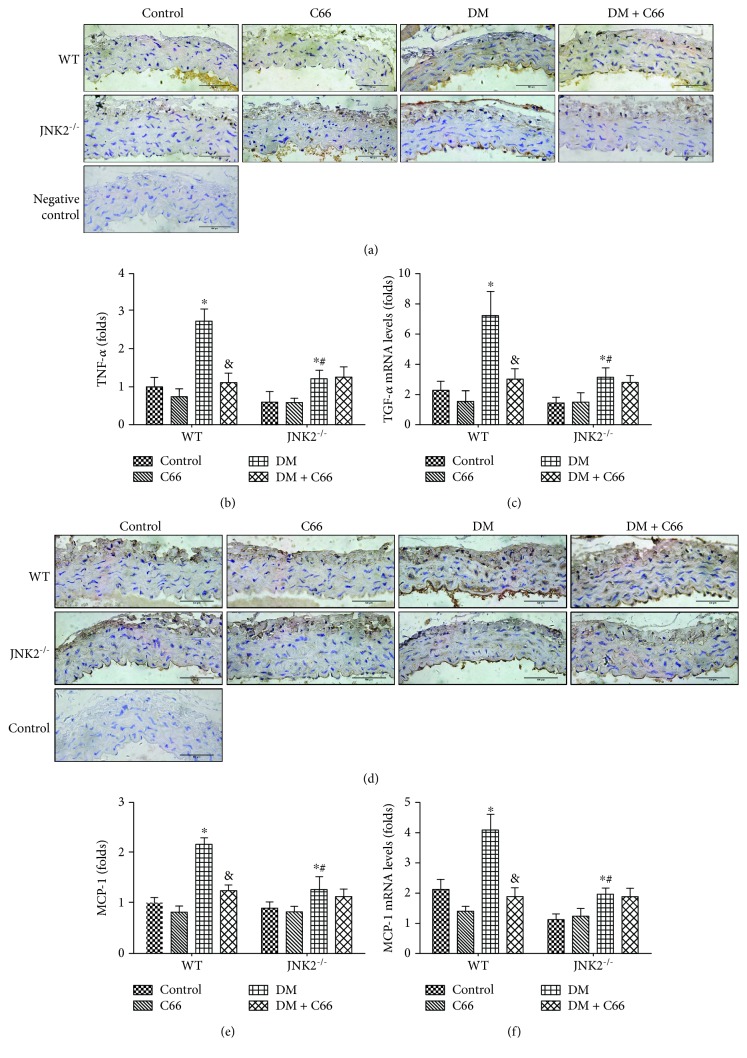
Protective effect of C66 or JNK2 deletion on diabetes-induced aortic inflammation. Immunohistochemical staining for the expression of TNF-*α* (a, b) and MCP-1 (d, e) and qPCR for the mRNA expression of TNF-*α* (c) and MCP-1 (f). *n* = 8; ^∗^ *P* < 0.05 DM vs. corresponding control group; ^&^ *P* < 0.05 DM + C66 vs. corresponding DM; ^#^ *P* < 0.05 JNK2^−/−^ mice vs. corresponding WT mice. DM: diabetes mellitus.

On account of the fact that both inflammation and oxidative damage are primary risk factors for the vascular endothelial remodeling, the expression of TNF-*α* (Figure 4(a)) and MCP-1 (Figure 4(d)) was examined with immunohistochemical staining, which showed a significant increase in aortic tunica media of diabetic mice, an effect that was completely prevented by C66 treatment or JNK2 deletion. Additionally, C66 was found to have no special effects on these inflammatory factors in JNK2^−/−^ DM mice (Figures 4(b) and 4(e)). The results of qPCR of TNF-*α* (Figure 4(c)) and MCP-1 (Figure 4(f)) were consistent with immunohistochemical staining.

**Figure 5:**
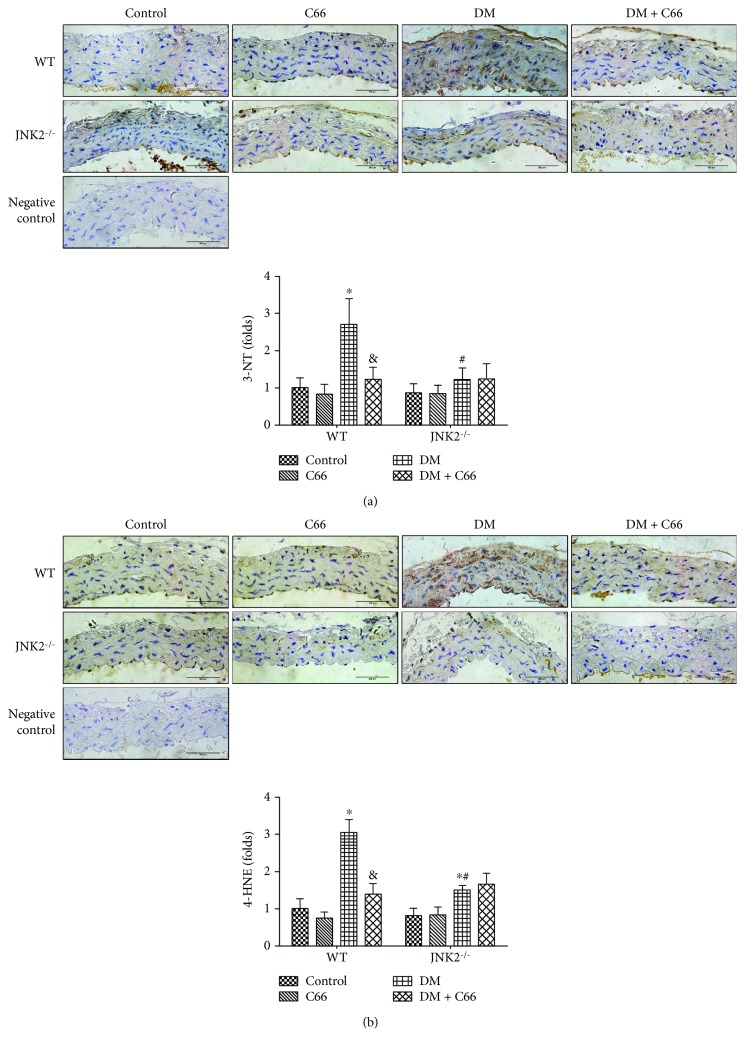
Protective effect of C66 or JNK2 deletion on diabetes-induced aortic oxidative stress. Immunohistochemical staining for the expression of 3-NT (a) and 4-HNE (b). *n* = 8; ^∗^ *P* < 0.05 DM vs. corresponding control group; ^&^ *P* < 0.05 DM + C66 vs. corresponding DM; ^#^ *P* < 0.05 JNK2^−/−^ mice vs. corresponding WT mice. DM: diabetes mellitus.

Considering that inflammation and oxidative stress are reciprocal cause and outcomes, 3-NT (Figure 5(a)) and 4-HNE (Figure 5(b)) were examined by immunohistochemical staining to evaluate oxidative and nitrative damage. The elevation of 3-NT and 4-HNE in the diabetic aorta can be significantly decreased by C66 or JNK2 deletion. However, C66 had no significant effect on 3-NT and 4-HNE in the aorta of JNK2^−/−^ diabetic mice.

### 3.4. The Expressions of Nrf2 and Its Downstream Genes

**Figure 6:**
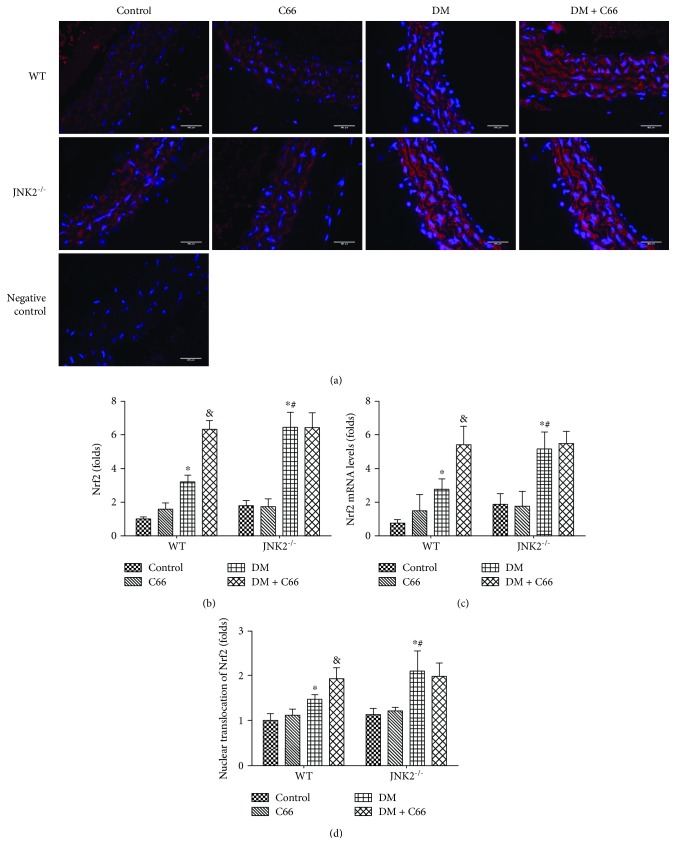
The expression of Nrf2 in the aortas. Aortic expression of Nrf2 was examined by immunofluorescent staining for its protein expression (red) (a, b) and real-time PCR for its mRNA level (c). The nuclear translocation of Nrf2 was also evaluated (d). *n* = 8; ^∗^ *P* < 0.05 DM vs. corresponding control group; ^&^ *P* < 0.05 DM + C66 vs. corresponding DM; ^#^ *P* < 0.05 JNK2^−/−^ mice vs. corresponding WT mice. DM: diabetes mellitus.

Oxidative stress has been extensively considered as a crucial mediator for various cardiovascular complications of diabetic patients. We assumed that the above pathological changes in the aortas of diabetic mice may predominantly attribute to the increased oxidative stress. The protective effect of C66 on diabetes-induced aortic pathogenesis may be mediated by upregulation of endogenous antioxidants. Our previous study showed that C66 can upregulate the expression of Nrf2 in diabetic mice [25]. In our present study, immunofluorescent staining showed that diabetics have slightly increased expression and nuclear translocation of Nrf2, which can be significantly increased by C66 treatment or JNK2 deletion (Figures 6(a), 6(b), and 6(d)). However, C66 treatment had no extra effect on Nrf2 expression in JNK2^−/−^ DM mice. The mRNA level of Nrf2 was consistent with immunofluorescent staining (Figure 6(c)).

**Figure 7:**
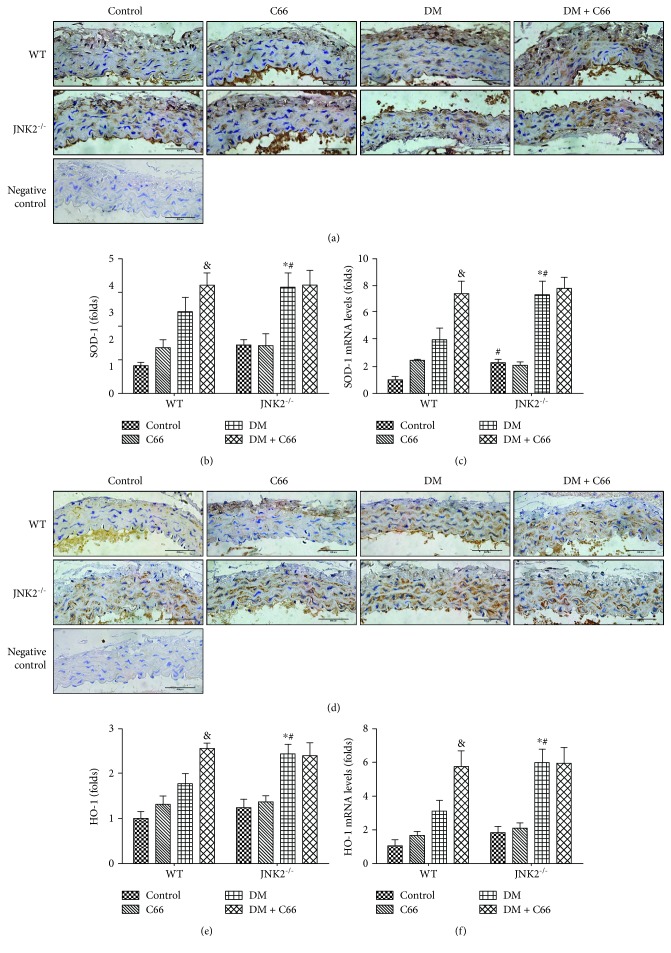
The expression of Nrf2 downstream genes. Immunohistochemical staining for the expression of SOD-1 (a, b) and HO-1 (d, e) and qPCR for the mRNA expression of SOD-1 (c) and HO-1 (f). *n* = 8; ^∗^ *P* < 0.05 DM vs. corresponding control group; ^&^ *P* < 0.05 DM + C66 vs. corresponding DM; ^#^ *P* < 0.05 JNK2^−/−^ mice vs. corresponding WT mice. DM: diabetes mellitus.

The Nrf2 downstream genes SOD-1 (Figures 7(a)–7(c)) and HO-1 (Figures 7(d)–7(f)) were also evaluated by immunohistochemical staining and qPCR. The results were consistent with Nrf2.

## 4. Discussion

We have provided the first evidence to show the significant protective effect of C66 on diabetes-induced aortic damage. In STZ-induced WT diabetic mice, significant increase in aortic oxidative damage, inflammation, apoptosis, and fibrosis has been found. All these pathogenic changes were obviously decreased by C66 treatment or JNK2 deletion. We also demonstrated that in the JNK2 deletion DM mice, the protective effect of C66 in the aorta cannot be revealed. These results suggest that C66 prevents diabetes-induced pathogenic changes in the aorta via inhibition of JNK2 function.

Inflammation plays a critical role in the development of diabetes and its complications. Chronic inflammation induces oxidative stress, apoptosis, endothelial dysfunction, and fibrosis, all of which contribute to tissue damage and the formation of new vascular structures [12, 25]. In our study, we showed the increased expressions of TNF-*α* and MCP-1 (Figure 4), as inflammation markers, in the aorta of the WT DM group, which was accompanied with increased expressions of markers of aortic fibrosis (CTGF, TGF-*β*1) (Figure 2), apoptosis (caspase-3) (Figure 3), and oxidative stress (3-NT, 4-HNE) (Figure 5) in the WT DM group. Either C66 treatment or JNK2 deletion can eliminate these increased expressions of markers in the aortas of diabetic mice. And C66 treatment was found no further effect on the JNK2^−/−^ DM group suggesting that the protection of C66 may target on JNK2 inhibition.

JNK, as a member of the mitogen-activated protein kinase family, regulates various cell stress responses, including inflammatory responses, oxidative stress, cell death, cell survival, and proteins expression [27]. In diabetes, obvious and sustained JNK activation is observed in different tissues [23, 25, 28, 29]. Thus, deregulating the activation of JNK is a potent therapeutic strategy for diabetes. Curcumin has shown to protect cardiovascular diseases via inhibition of JNK [30, 31]. In the previous studies from our team, C66, as a novel curcumin analogue, has also been revealed significant effect on inhibition of JNK [23, 25]. Moreover, Pan et al. have reported that C66 exhibits a high JNK2-binding affinity in a molecular docking, which leads to its anti-inflammatory actions [26]. In our present study, we have demonstrated that JNK2 deletion indeed alleviated diabetes-induced aortic inflammation, oxidative stress, apoptosis, and fibrosis, but there was no obvious effect in the JNK2^−/−^ diabetic mice aorta after C66 treatment. These results suggest that the protection of C66 on diabetes-induced aortic damage may depend on JNK2 suppression.

Oxidative stress is involved in the pathogenesis of diabetes-induced cardiovascular changes, and hyperglycemia is the causal link between diabetes and increased oxidative stress [32, 33]. Excessive ROS generation has been identified as an initial pathogenic factor of diabetic aortic damage. Hence, enhanced endogenous antioxidative capacity has been considered effective in attenuating these damages. Nrf2 regulates multiple adaptive responses to oxidative stress and is also involved in cell migration, proliferation, apoptosis, and differentiation [34]. And Nrf2 has been shown therapeutic effects in diabetic complications by contributing to the inducible expression of antioxidant enzymes. Nrf2 silencing has been verified to inhibit the migration, proliferation, and secretion of endothelial progenitor cells, but increases oxidative stress and cell senescence [35]. Furthermore, overexpression of Nrf2 inhibits ROS and inflammatory cytokine expression in the high glucose-cultured endothelial progenitor cells [36]. As a downstream factor of JNK, we speculated that Nrf2 may involve in the C66 protection. Here, we verified that diabetes can slightly increase Nrf2 expression and function, which were reflected by HO-1 and SOD-1. It is suggested that at certain early stages, Nrf2 acts as a protective mechanism attempting to protect the tissue, which was consistent with our previous research [25]. In the present study, a mild increase in Nrf2 expression and function may remain not enough to compensate the severe damage induced by diabetes. Either C66 treatment or JNK2 deletion effectively increased Nrf2 expression and function (Figures 6 and 7). However, the JNK2^−/−^ DM + C66 group has not shown significant increase of Nrf2 expression and its function, compared with the JNK2^−/−^ DM group. It indicated that JNK2 may be the key factor that regulates Nrf2 and its function in the protection of C66.

In conclusion, we have investigated that the protective effect of C66 in the diabetic aorta mainly depends on JNK2 inactivation. Either C66 treatment or JNK2 deletion can reverse and/or prevent the progression of diabetes-induced aortic inflammation, oxidative damage, apoptosis, and fibrosis. Mechanism responsible for this protective effect of C66 is mediated by inhibition of JNK2 that may be related to upregulation of Nrf2 expression and function.

## Figures and Tables

**Table 1:** Primer sequences for real-time quantitative PCR.

Gene	Forward primer	Reverse primer
CTGF	GGGCCTCTTCTGCGATTTC	ATCCAGGCAAGTGCATTGGTA
TGF-*β*1	CTCCCGTGGCTTCTAGTGC	GCCTTAGTTTGGACAGGATCTG
MCP-1	TTAAAAACCTGGATCGGAACCAA	GCATTAGCTTCAGATTTACGGGT
TNF-*α*	CCCTCACACTCAGATCATCTTCT	GCTACGACGTGGGCTACAG
HO-1	AAGCCGAGAATGCTGAGTTCA	GCCGTGTAGATATGGTACAAGGA
SOD-1	AACCAGTTGTGTTGTCAGGAC	CCACCATGTTTCTTAGAGTGAGG
Nrf2	CTTTAGTCAGCGACAGAAGGAC	AGGCATCTTGTTTGGGAATGTG

## Data Availability

The data used to support the findings of this study are included within the article.

## Supplementary Materials

Supplementary materials Supplementary Figure 1. The blood glucose level of each group at the beginning of the experiment. *n* = 8; ^∗^ *P* < 0.05 vs. corresponding control group. Supplementary Figure 2. The hematoxylin and eosin staining of the aortas. And the wall intima thickness was evaluated. *n* = 8; ^∗^ *P* < 0.05 vs. corresponding control group.
