# Supplementary material for: Novel Curcumin C66 That Protects Diabetes-Induced Aortic Damage Was Associated with Suppressing JNK2 and Upregulating Nrf2 Expression and Function
Source: Oxid Med Cell Longev. 2018 Nov 28;2018:5783239. doi: 10.1155/2018/5783239 (PMC6304198; doi:10.1155/2018/5783239)
Supplement: Supplementary materials — Supplementary Figure 1. The blood glucose level of each group at the beginning of the experiment. n = 8; ∗ P < 0.05 vs. corresponding control group. Supplementary Figure 2. The hematoxylin and eosin staining of the aortas. And the wall intima thickness was evaluated. n = 8; ∗ P < 0.05 vs. corresponding control group. [file 5783239.f1.docx]

**Supplementary Materials**

**
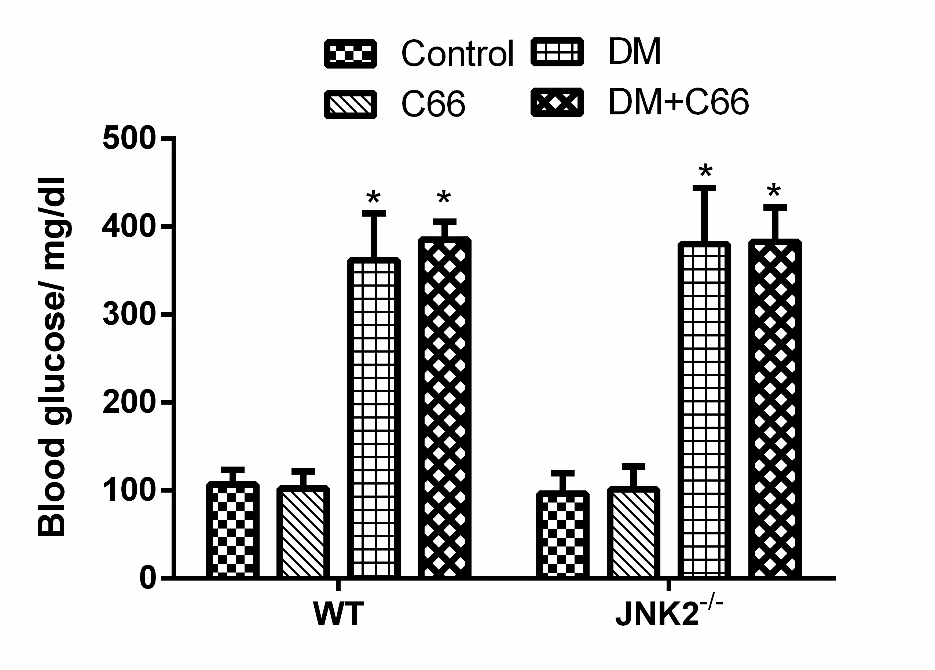
**

**Supplementary Figure 1.** The blood glucose level of each group at the beginning of the experiment.

n=8; **P<0.*05 *vs.* corresponding control group.


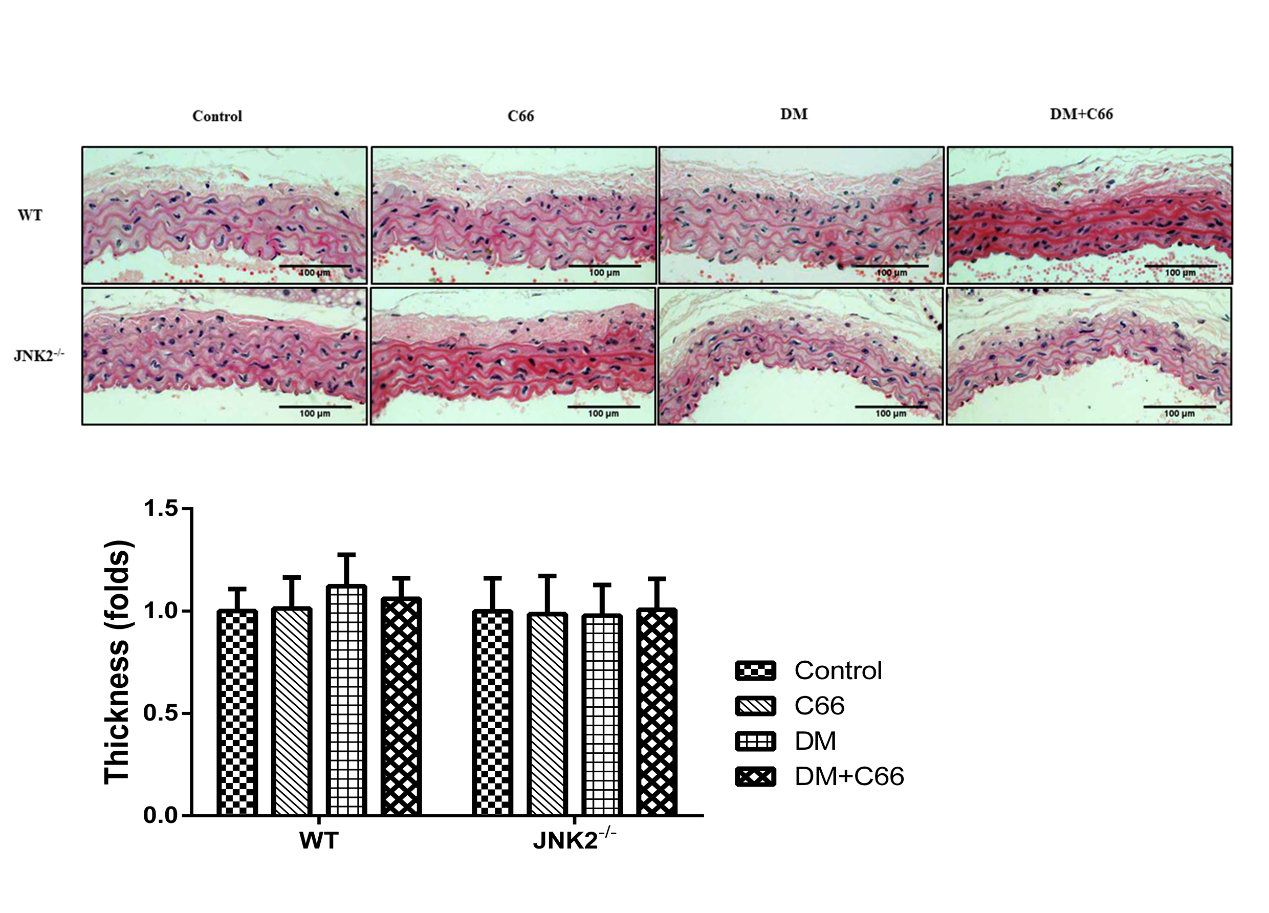


**Supplementary Figure 2.** The haematoxylin and eosin staining of aortas. And the wall intima thickness were evaluated.

n=8; **P<0.*05 *vs.* corresponding control group.
